# Use of Surface Plasmon Resonance Technique for Studies of Inter-domain Interactions in Ion Channels

**DOI:** 10.1007/978-1-0716-3818-7_7

**Published:** 2024

**Authors:** Purushottam B Tiwari, Pareesa Kamgar-Dayhoff, Prakriti Tiwari, Maria I McKillop, Tinatin I Brelidze

**Affiliations:** 1 Department of Oncology, Georgetown University Medical Center, Washington, DC, USA.; 2 Department of Pharmacology and Physiology, Georgetown University Medical Center, Washington, DC, USA.; 3 Department of Biology, College of Arts & Sciences, Georgetown University, Washington, DC, USA.

**Keywords:** SPR, Potassium channels, Ion channel gating, Ion channel function, KCNH, EAG, hERG, Kv10.1, Kv11.1

## Abstract

Ion channels are transmembrane proteins essential for cellular functions and are important drug targets. Surface plasmon resonance (SPR) is a powerful technique for investigating protein–protein and protein–small molecule ligand interactions. SPR has been underutilized for studies of ion channels, even though it could provide a wealth of information on the mechanisms of ion channel regulation and aid in ion channel drug discovery. Here we provide a detailed description of the use of SPR technology for investigating inter-domain interactions in KCNH potassium-selective and voltage-gated ion channels.

## 1 Introduction

**Fig. 1:**
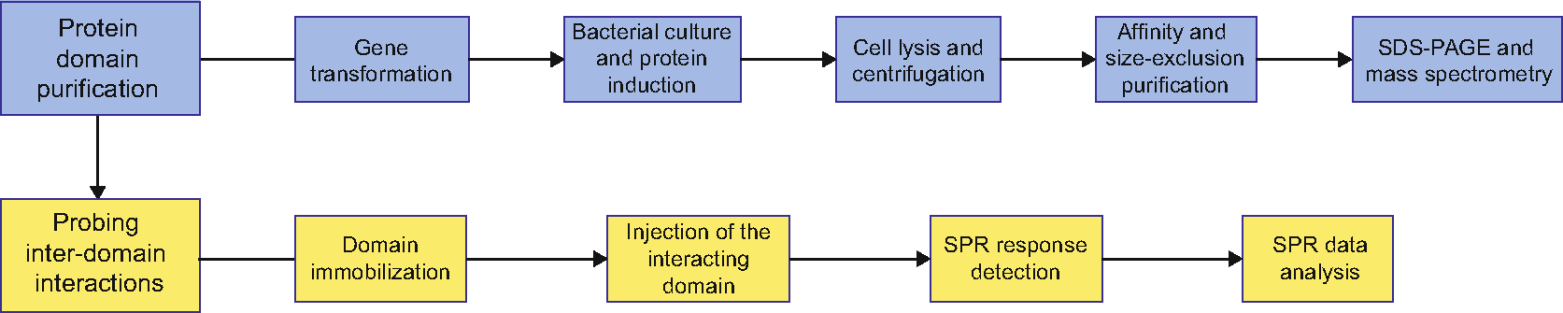
Flowchart for protein domain purification and SPR experiments for probing inter-domain interactions

Ion channels contain multiple domains with diverse functions. For instance, the pore domain provides passageways for ions to cross the membrane down their electrochemical gradient, while the voltage-sensing domain detects changes in membrane potential. Many ion channels are also sensitive to ligands and contain specialized ligand-binding domains. Inter-domain and ligand–ion channel interactions are essential for channel opening and closing (gating). Observing the changes in the inter-domain interactions in the absence and presence of ligands in unmodified full-length channels during gating in their native environment is very challenging, if not impossible, at the present state of the field. Therefore, probing the inter-domain interactions and small molecule ligand (SML)–ion channel interactions under more stripped-down conditions with a variety of complementary methods, including structural, electrophysiology, and fluorescence-based approaches, is essential to gain the understanding of ion channel gating mechanisms. Surface plasmon resonance (SPR) is one of the techniques that offers quantitative assessment of the inter-domain interactions and direct SML binding to ion channels that could be further integrated with findings uncovered with other methods. SPR is especially well suited for studies of ion channels with soluble domains that can be easily purified in isolation. As the full-length ion channel purification becomes more mainstream, the application of SPR technique to probing inter-domain and SML–ion channel interactions could be extended to full-length ion channels as well. Here we describe the application of SPR for investigating inter-domain interactions in KCNH potassium-selective and voltage-gated ion channels (the flowchart of steps involved in this approach is shown in Fig. 1).

**Fig. 2:**
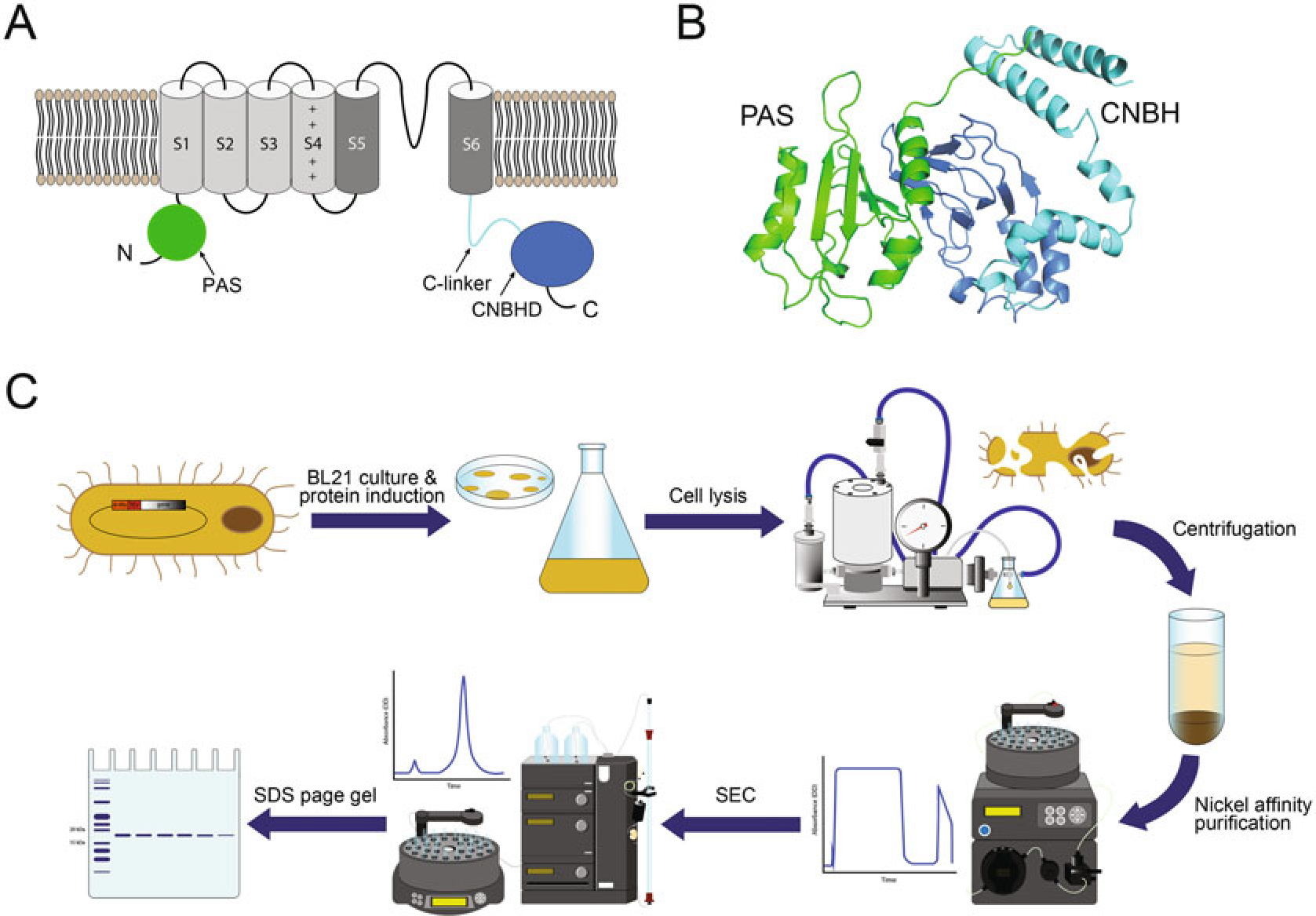
KCNH channel membrane topology and schematics of PAS and CNBH domain purification. (**a**) Cartoon of a KCNH channel subunit. The PAS domain is green, the transmembrane segments S1–S4 forming the voltage-sensor domain are light gray, the transmembrane domains S5–S6 forming the pore domain are dark gray, the C-linker is cyan, and CNBH is dark blue. (**b**) The ribbon representations of the PAS and CNBH domains from the adjacent subunits of KCNH2 channels (PDB ID: 5VA2). (**c**) Graphic summary of experimental steps involved in the purification of the isolated PAS and CNBH domains of KCNH channels

Similar to other potassium channels, KCNH channels are assembled from four subunits. Each subunit contains an intracellular N-terminal Per-Arnt-Sim (PAS) domain, six transmembrane segments (S1–S6), and an intracellular C-terminal cyclic nucleotide-binding homology (CNBH) domain linked to S6 via the C-linker (*see* Fig. 2a) [1–3]. The transmembrane segments are further subdivided into the voltage-sensor domains formed by S1–S4 segments and centrally located pore domain formed by S5–S6 from all four subunits. The PAS domains interact with the CNBH domains from adjacent subunits (*see* Fig. 2b), and these inter-domain interactions confer many of the functional properties of KCNH channels, including the kinetics of deactivation in KCNH2 channels [3–6]. In addition, PAS and CNBH domains can function as ligand-binding domains in KCNH channels [7–10]. The PAS and CNBH domains of KCNH channels can be purified as isolated proteins, as illustrated in Fig. 2c and described in detail in the Protein Sample Preparation section. The structure of the isolated PAS and CNBH domains and the domains in the intact full-length KCNH channels is very similar [2, 3, 11–15]. Therefore, the findings of SPR-based studies on the isolated PAS and CNBH domains have high relevance for the intact full-length channels.

**Fig. 3:**
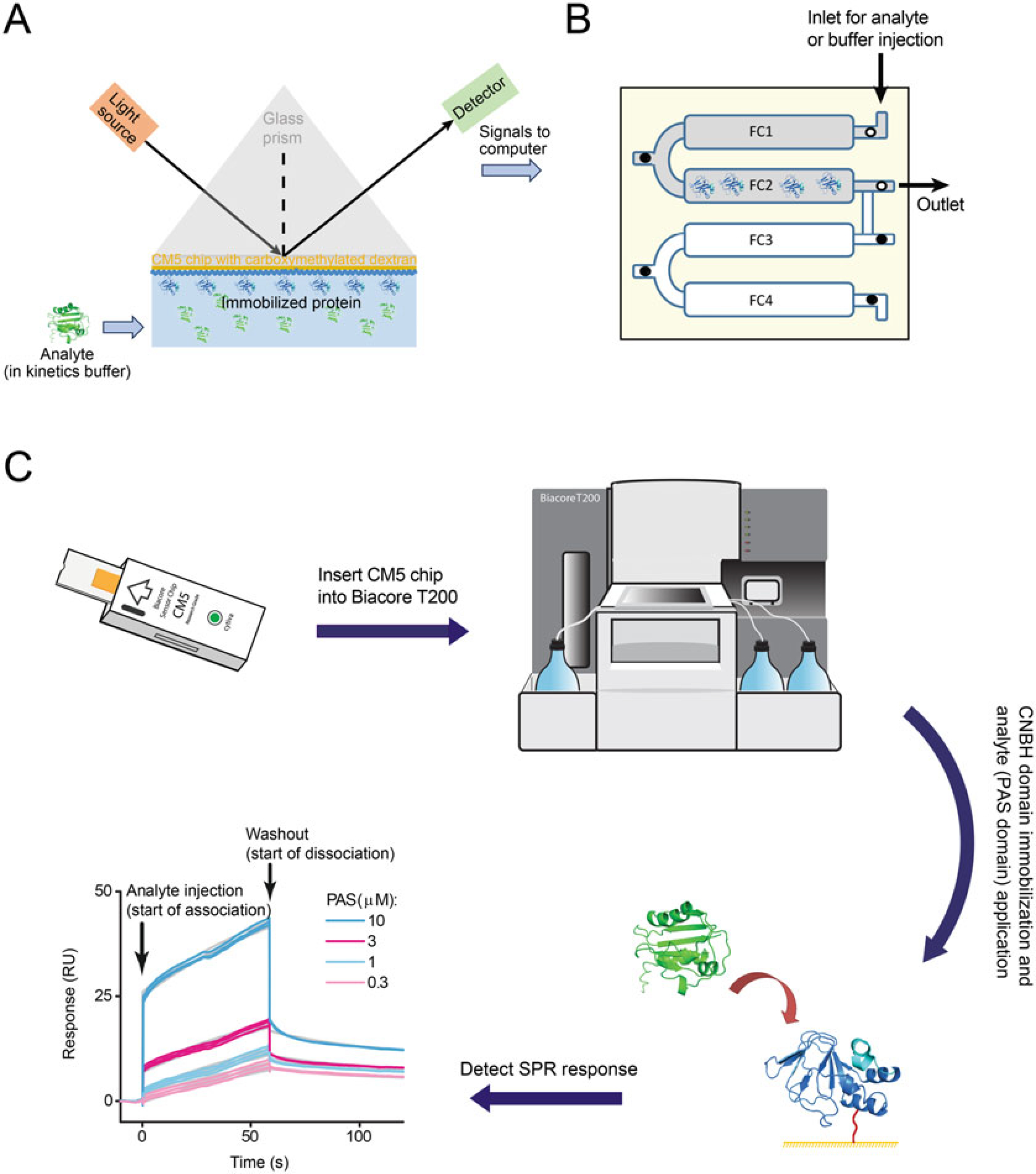
Schematics of the SPR technique and experiment. (**a**) Schematic of the SPR technique, indicating the incident and reflected light off the sensor chip surface with CNBH domains immobilized on the chip surface and PAS domains applied as free analytes. (**b**) FC arrangement and fluidics of the CM5 sensor chip with the CNBH domain immobilized on FC2 and no protein applied on FC1 used as a reference FC. White circles indicate open valves and black circles indicate closed valves. (**c**) Graphic summary of experimental steps involved in the SPR signal detection for the PAS and CNBH domain binding. The gray lines in the SPR sensorgrams represent fits of the SPR data with the two-state reaction binding model using the Biaevaluation software with the K_d_ value of 1.4 μM

The SPR technique is frequently used for investigating protein–protein and SML–protein interactions [16–18]. We have used SPR technique to probe interactions between the isolated PAS and CNBH domains of KCNH channels [15], to identify novel SMLs that bind to these domains using chemical library screening [8], and to further characterize SML–ion channel interactions [8, 9]. SPR technique detects changes in the refractive index for the chip surface with immobilized protein upon binding of a binding partner. In this technology, a metal-coated (typically gold) glass slide is used as a sensor chip. The sensor chip can be modified and activated to immobilize proteins using various surface chemistries [19, 20]. The protein (or other biomolecules) immobilized onto the chip surface is termed as a “ligand” in the SPR technique, and the binding partner, which could be a protein, SML, DNA, or RNA, that flows over the ligand-immobilized surface in solution is called an “analyte” [21]. Since in the ion channel field “ligand” frequently refers to a small molecule binder of ion channels, to avoid the confusion, we will be referring to the immobilized binding partner as an immobilized protein instead of a ligand. Figure 3a, b shows a schematic of the optical and fluidic systems used in the SPR technology. Analyte–ligand binding changes the mass on the surface and the refractive index in the vicinity of the metal surface with the immobilized ligand [22–25]. The change in the refractive index is detected as SPR response, with plots of SPR response over time called SPR sensorgrams [26]. The SPR technique detects real-time interactions between two binding partners [27–29]. This technique is especially well-suited for the studies of inter-domain interactions in ion channels because no additional modification of the interacting domains is needed for the use with SPR. In addition, relatively low amounts of the protein sample are sufficient for inter-domain binding detection and determining the affinity of the binding using SPR [30]. Below we provide a detailed description of the SPR-based experiments to probe interactions between the PAS and CNBH domains of KCNH2 channels, also known as ether--á-go-go-related gene (hERG) and Kv11.1 channels.

## 2 Materials

### 2.1 Protein Sample Preparation

1. cDNA encoding the PAS domain of KCNH2 channels (GI number 7531135, amino acids 2–134) subcloned into pETM11 bacterial expression vector (*see* Note 1).
2. cDNA encoding the C-linker/CNBH domain of KCNH2 channels, referred as CNBH domain for simplicity (GI number 7531135, amino acids 734–867), subcloned into pETM11 bacterial expression vector.
3. BL21 (DE3) cells.
4. Buffer 1: 150 mM KCl, 1 mM tris(2-carboxyethyl)phosphine (TCEP), 10% glycerol, and 30 mM HEPES, pH 7.5 (*see* Note 2).
5. Isopropyl β-D-1-thiogalactopyranoside (IPTG).
6. 4-(2-Aminoethyl)benzenesulfonyl fluoride hydrochloride (AEBSF).
7. DNase I.
8. Imidazole.
9. Column for purifying proteins with immobilized metal ion affinity chromatography.
10. Size exclusion column.
11. SDS-PAGE gel (12% Bis-Tris).
12. Coomassie Brilliant Blue.

### 2.2 Protein Sample Dilutions

1. 96-deep-well plate.
2. Tween 20.
3. Buffer 1: 150 mM KCl, 1 mM TCEP, 10% glycerol, and 30 mM HEPES, pH 7.5.
4. Buffer 2: 150 mM KCl, 1 mM TCEP, 10% glycerol, 0.05% (v/v) Tween 20, and 30 mM HEPES, pH 7.5 (*see* Note 3).

### 2.3 Buffers for Protein Immobilization and SPR Experiments

1. Immobilization buffer: 10 mM sodium acetate, pH 5.5.
2. Immobilization running buffer: 150 mM NaCl, 10 mM HEPES, 0.05% (v/v) surfactant P20, pH 7.4.
3. Kinetics running buffer (the same as Buffer 2): 150 mM KCl, 1 mM TCEP, 10% glycerol, 0.05% (v/v) Tween 20, and 30 mM HEPES, pH 7.5.

### 2.4 Protein Immobilization

1. SPR equipment (*see* Note 4).
2. CM5 sensor chip.
3. Immobilization buffer (see above).
4. Immobilization running buffer (see above).
5. 7 mM plastic vial and seal with rubber cap.
6. 160 uL 1 M ethanolamine (*see* Note 5).
7. N-Hydroxysuccinimide (NHS).
8. 1-Ethyl-3-(3-dimethylaminopropyl)carbodiimide hydrochloride (EDC).

### 2.5 SPR Response Detection

1. Rack tray to handle sample solutions.
2. Glycine pH 2.0.
3. Opto-fluidic system to deliver solutions to chip surface and to monitor change in refractive index on the sensor surface (*see* Note 6). A schematic of opto-fluidic system and the steps for the SPR signal detection are shown in Fig. 3a–c.
4. Control software to run immobilization and kinetics (*see* Note 7).
5. Evaluation software for analysis (*see* Note 7).

## 3 Methods

### 3.1 Protein Sample Preparation

1. Grow bacterial cultures transformed with the pETM11 vector containing the PAS or CNBH domains at 37 °C.
2. At OD of 0.6–0.8, induce PAS and CNBH domain expression with 1 mM IPTG.
3. Continue growing bacterial cultures overnight at 18 °C.
4. Harvest bacteria by centrifugation at 5000 rpm for 15 min at 4 °C (*see* Note 8).
5. Resuspend the bacterial cell pellet in Buffer 1 supplemented with 1 mM AEBSF and 2.5 mg/mL DNase I.
6. Lyse the cells with a homogenizer (*see* Note 9).
7. Separate soluble protein by centrifugation at 30,000 rpm for 1 h at 4 °C.
8. Load the supernatant containing the soluble protein onto an affinity column, and use Ni^2+^–nitrilotriacetic acid (NTA) chromatography to purify PAS and CNBH domains containing the 6-His tag.
9. Wash the affinity column with Buffer 1 and elute the protein with Buffer 1 supplemented with 500 mM imidazole.
10. Cleave the 6xHis tag with TEV protease (*see* Note 10).
11. Equilibrate a size exclusion column with Buffer 1, and further purify the PAS and CNBH domains in Buffer 1 using size exclusion chromatography (*see* Notes 11 and 12).
12. Elute the PAS and CNBH domains off the size exclusion column in 500 μL fractions. The fractions containing the domains are initially identified based on the elution profile.
13. Verify the molecular weight of the purified protein, and evaluate the purity of the protein sample with Coomassie Brilliant Blue-stained SDS-PAGE gels (*see* Note 13).
14. Mix the elusion fractions containing the PAS domains, and aliquot them in 150 μL volumes. Since the PAS domain will be used as an analyte, these volumes are appropriate for preparing dilutions with different concentrations of the analyte for injections over the immobilized CNBH domains.
15. Mix elusion fractions containing the CNBH domains, and aliquot them in 20–25 μL volumes. Since the CNBH domain will be immobilized on the SPR chip, smaller volumes of the sample are sufficient for immobilization.
16. Determine the concentration of the purified PAS and CNBH domains using Bradford protein assay. The protein concentration after the size exclusion purification is usually sufficient for the SPR experiments.
17. Determine the exact weight of the aliquoted PAS and CNBH domains with mass spectrometry (electrospray).
18. Store the purified PAS and CNBH domains at −80 °C i n the small aliquots before use (*see* Note 14).

### 3.2 Protein Sample Dilutions

1. Thaw PAS domain aliquots and add 0.05% (v/v) Tween 20. For instance, add 4 μL of 10% Tween 20 to 800 μL of the PAS domain aliquots (*see* Note 15).
2. Prepare PAS domain dilutions at (in μM) 10, 3, 1, 0.3, and 0 concentrations in kinetics running buffer (Buffer 2). The volume for each concentration should be 330 μL.
3. For the serial protein dilutions, it is important to make sure that enough aliquots are thawed to have sufficient volume of the highest protein concentration (10 μM) for the subsequent dilutions (*see* Notes 16 and 17). An example of the dilutions for the PAS domain aliquots purified at 50 μM concentration is shown below. While the required volume for each analyte concentration is only 330 μL, the calculations below include extra volumes to accommodate the serial dilutions and also make pipetting easier by using round numbers for volume measurements.
4. Transfer Buffer 2 and the analyte dilutions into the 96-deep-well plate so that Buffer 2 will be injected over the immobilized protein first, followed by the injection of the analytes from low to high concentrations. A typical arrangement would be Buffer 2 in well A1, with PAS concentrations of 0.3 μM to 10 μM in wells A2–A5, respectively. The volume in each well should be 330 μL.

**Table:** 

Final PAS concentration	Volume of the aliquots	Buffer 2	Total volume
10 μM	150 μL of PAS aliquots	600 μL	750 μL
3 μM	180 μL of 10 μM PAS	420 μL	600 μL
1 μM	50 μL of 10 μM PAS	450 μL	500 μL
0.3 μM	180 μL of 1 μM PAS	420 μL	600 μL

### 3.3 Protein Immobilization

1. Dissolve the proteins to be immobilized (CNBH domains) in the immobilization buffer in a 7 mM plastic vial, and seal with the rubber cap.
2. Place the sealed NHS–EDC, protein, and ethanolamine in the sample rack tray, and insert into the machine (*see* Note 18).
3. CM5 chip has four flow cells (FCs) (*see* Fig. 3b). Activate CM5 chip surface by injecting NHS–EDC solution onto two neighboring FCs, for example, FC1 and FC2, for 720 s (*see* Note 19).
4. Inject protein onto FC2 until desired protein immobilization level is roughly achieved, e.g., for 300 s, while leaving FC1 free of protein injections.
5. Inject ethanolamine solution into FC1 and FC2 for 720 s. This blocks any remaining active surface sites. Here FC1 works as the reference FC for the active FC (FC2) with immobilized proteins onto it.
6. Determine the immobilized protein level by subtracting the signal level just before injection of protein from the signal level after ethanolamine injection is completed. This difference is used to determine the density of the immobilized protein in response units (RU, 1 RU = 1 pg of protein per mm^2^) [31]. For the SPR experiments described here, we immobilized ~7000 RU of the CNBH domain of KCNH2 channels.
7. The immobilization running buffer should be running in the background during protein immobilization.

### 3.4 SPR Response Detection

1. Inject analyte solutions (PAS solutions) at different concentrations at the flow rate of 50 μL/min into both FC1 and FC2 in the kinetics running buffer using the association and dissociation times of 60 s and 150 s, respectively (*see* Notes 20 and 21). Make sure to run kinetics buffer-only solution as a blank.
2. Run the kinetics running buffer in the background.
3. Run the method so that the software records data in the format of FC2–FC1. In this form of data collection, the signals corresponding to the reference FC (FC1) are subtracted from the response detected in the active FC (FC2).
4. Inject analyte and blank solutions in three technical replicates in one experiment (we recommend at least duplicate).
5. Inject regeneration solution (in this case glycine pH 2.0) for 15 s at the end of dissociation to remove any remaining analytes on the surface before another cycle of injection of analyte solution starts (*see* Note 22).
6. The sensorgrams recorded using the control software can be opened in evaluation software for data analysis. The signals for analysis should be both reference (signals from FC1) and blank (signals contributed by blank) subtracted.
7. Each experiment should be repeated at least three times on three different sensor chips. Therefore, the number of protein aliquots should be determined accordingly. Frequently, it is necessary to purify the domains several times to have sufficient protein to repeat the SPR experiments three times.

### 3.5 SPR Data Analysis

1. Use an evaluation software (*see* Note 23) for the initial data analysis and plotting. The sensorgrams can either be copied from the evaluation software window or raw data can be exported from the evaluation software and replotted using any other plotting software for the subsequent analysis, if desired (*see* Note 24).
2. Check if the data can be fitted using a steady-state affinity fitting. If there are very fast association and dissociation phases and the binding reached a plateau, this method can be used [22].
3. If data cannot be fitted using the steady-state binding model, then check whether 1:1 monophasic binding can be used to fit the kinetics data to determine binding parameters, such as the association rate constant (k_a_), dissociation rate constant (k_d_), and equilibrium dissociation constant (K_d_).
4. If data cannot be fitted using 1:1 kinetics binding model, then check which biphasic model can better represent the experimental data. We recommend identifying the correct underlying biphasic binding mechanism as described in the prior publications [32, 33].
5. For the KCNH2 PAS and CNBH domain interactions considered here, the two-step conformational change model was determined as the best model to better predict the experimental data based on the prior publication [32], and this model was used to fit the data to determine the K_d_ value (*see* Note 25) [15].

### 3.6 Conclusions

Here we described the application of the SPR technique to investigating PAS and CNBH domain interactions in KCNH2 channels. This approach could be used to investigate the interactions between PAS and CNBH domains both in the absence and presence of SMLs. Importantly, many disease-associated mutations in KCNH channels are located at the PAS and CNBH domain interaction interface [2, 5, 34, 35], and the SPR technique could also be used to investigate the effect of the disease-associated mutations on the PAS and CNBH domain interactions, as we reported before [15].

The SPR technique requires protein immobilization on the sensor chip surface. This could affect the conformation of the immobilized protein and its interactions with binding partners. It is important to keep this limitation in mind when interpreting the results of the SPR experiments. To determine if the immobilization influences interactions detected with SPR, one could consider repeating the experiments using different immobilization chemistry, e.g., immobilization on the NTA sensor chip using Ni^2+^–NTA and the 6-His tag coupling, using a CM5 chip and anti-His antibody capture method, using different chips such as CM4 and C1 chips, or reversing the analyte and immobilized protein (e.g., immobilizing PAS domains and using CNBH domains as analytes).

## 4 Notes

1. PAS and CNBH genes are cloned into the pETM11 bacterial expression vector with an N-terminal 6-His affinity tag followed by a tobacco etch virus (TEV) protease cleavage site.
2. All buffer solutions are prepared using deionized ultrapure water, filtered and stored in sterile 500 mL or 1 L filter storage bottles at 4 °C for no longer than 3–4 days.
3. To avoid using volumes that are too small, first make 10% (v/v) Tween 20 by mixing 2 mL of Tween 20 with 18 mL Buffer 1. Then add 10% (v/v) Tween 20 to Buffer 1 to make Buffer 2, e.g., add 10 mL of 10% (v/v) Tween 20 to 2 L Buffer 1. For increased accuracy, one might wish to take out 10 ml from 2 L of Buffer 1 prior to adding 10 mL of 10% (v/v) Tween 20. However, then the analyte (PAS protein) dilutions have to be prepared the same way to avoid a mismatch between the kinetics running buffer and analyte dilutions.
4. We use Biacore T200 (GE Healthcare, now Cytiva) for our experiments.
5. We use amine coupling kit from Cytiva that contains ethanolamine, NHS, and EDC.
6. The opto-fluidic system is embedded within the Biacore T200 unit that we use in our experiments.
7. A software to run the SPR instrument and to acquire and analyze the SPR data is supplied with the Biacore T200 as an accessory.
8. The bacterial pellet can be stored at −80 °C prior to the purification with the affinity chromatography.
9. We use EmulsiFlex-C5 homogenizer (Avestin) to lyse the cells.
10. Cleavage of the 6-His tag is optional since proteins will be immobilized using amine chemistry that does not require the 6-His tag for immobilization. The cleaved 6-His tag can be purified away from the PAS and CNBH domains with the size exclusion chromatography.
11. We use a Superdex 200 Increase column (Cytiva) for purifying the PAS and CNBH domains with the size exclusion chromatography (SEC).
12. To increase protein purity, always use SEC as the last step for the PAS and CNBH domain purification, even if the 6-His tag cleavage is not required.
13. The protein samples need to be pure when inspected on the Coomassie gel as impurities may interfere with the inter-domain interactions.
14. Having 10% glycerol in the solution increases PAS and CNBH domain stability during the transition between freezing for the storage at −80 °C and thawing for the SPR experiments conducted at 25 °C.
15. Keep protein solutions on ice while making the dilutions.
16. It is important to determine the smallest volume of aliquots needed for the experiment, but also it is best to choose round volumes that are easy to handle, e.g., avoid using 200.4 μL and instead find out how to make dilutions for 200 μL.
17. We recommend using the same solution for the kinetics running buffer and analyte dilutions to avoid any possible mismatch that could be registered as a false SPR signal.
18. It is important to follow manufacturer’s instructions on how to dissolve NHS and EDC. Make aliquots of each and freeze until use. Thaw at 4 °C.
19. Since the CM5 chip has four FCs, even after reserving one flow cell as a control, three different proteins can be immobilized on the same sensor chip, and binding interactions of these three different proteins with analytes could be detected in one experiment.
20. Biacore control software offers to write methods to inject analyte and regeneration solutions automatically. The association time (contact time) and dissociation time can be easily changed by the user. Here association time refers to the duration within which the injected analyte binds gradually to the immobilized protein on the chip surface, and dissociation time refers to the duration within which analyte injection is stopped (at the beginning of dissociation) and buffer only breaks the analyte–ligand complex.
21. While here we are using only one analyte, binding of a few more (typically up to five) different analytes applied over the range of concentrations can be tested for the same immobilized domains in the same experiment, as long as the immobilized protein is active on the surface. With time, the level of protein immobilization decreases. Therefore, examining more than five different analytes in the same experiment may not be feasible.
22. Appropriate regeneration solution is selected such that it breaks analyte–ligand complex without affecting the activity of proteins immobilized onto the chip surface. We recommend to start testing with weaker solutions such as 1 M NaCl.
23. We used Biaevaluation software version 1.0.
24. We use Origin software for replotting the SPR data; however, other commonly used software such as GraphPad Prism or Igor Pro can also be used.
25. All fitting models described here plus a few other biphasic models are included in the Biaevaluation software for data analysis.
